# Artificial Intelligence for Predicting Microsatellite Instability Based on Tumor Histomorphology: A Systematic Review

**DOI:** 10.3390/ijms23052462

**Published:** 2022-02-23

**Authors:** Ji Hyun Park, Eun Young Kim, Claudio Luchini, Albino Eccher, Kalthoum Tizaoui, Jae Il Shin, Beom Jin Lim

**Affiliations:** 1 Department of Pathology, Yonsei University College of Medicine, Seoul 03722, Korea; eiodkeiod@naver.com; 2 Evidence-Based and Clinical Research Laboratory, Department of Health, Social and Clinical Pharmacy, College of Pharmacy, Chung-Ang University, Seoul 06974, Korea; eykimjcb777@cau.ac.kr; 3 Department of Diagnostics and Public Health, Section of Pathology, University of Verona, 37134 Verona, Italy; claudio.luchini@univr.it; 4 ARC-Net Research Center, University and Hospital Trust of Verona, 37134 Verona, Italy; 5 Department of Pathology and Diagnostics, University and Hospital Trust of Verona, 37134 Verona, Italy; albino.eccher@aovr.veneto.it; 6 Laboratory of Microorganisms and Active Biomolecules, Sciences Faculty of Tunis, Tunis El Manar University, Tunis 2092, Tunisia; kalttizaoui@gmail.com; 7 Department of Pediatrics, Yonsei University College of Medicine, Seoul 03722, Korea; 8 Department of Pathology, Gangnam Severance Hospital, Yonsei University College of Medicine, Seoul 06273, Korea

**Keywords:** microsatellite instability, DNA mismatch repair, digital pathology, deep learning, artificial intelligence

## Abstract

Microsatellite instability (MSI)/defective DNA mismatch repair (dMMR) is receiving more attention as a biomarker for eligibility for immune checkpoint inhibitors in advanced diseases. However, due to high costs and resource limitations, MSI/dMMR testing is not widely performed. Some attempts are in progress to predict MSI/dMMR status through histomorphological features on H&E slides using artificial intelligence (AI) technology. In this study, the potential predictive role of this new methodology was reviewed through a systematic review. Studies up to September 2021 were searched through PubMed and Embase database searches. The design and results of each study were summarized, and the risk of bias for each study was evaluated. For colorectal cancer, AI-based systems showed excellent performance with the highest standard of 0.972; for gastric and endometrial cancers they showed a relatively low but satisfactory performance, with the highest standard of 0.81 and 0.82, respectively. However, analyzing the risk of bias, most studies were evaluated at high-risk. AI-based systems showed a high potential in predicting the MSI/dMMR status of different cancer types, and particularly of colorectal cancers. Therefore, a confirmation test should be required only for the results that are positive in the AI test.

## 1. Introduction

Microsatellites, also called Short Tandem Repeats (STR) or Simple Sequence Repeats (SSR) are short, repeated sequences of 1–6 nucleotides present throughout the genome [1]. Their repeated natures make them particularly vulnerable to DNA mismatch errors (insertion, deletion, and misincorporation of base) that occur during DNA replication and recombination. One of the most important DNA repair systems, called mismatch repair (MMR), usually correct these errors in normal tissues. However, in cases of alterations of MMR genes (called defective MMR; dMMR), namely MLH1, PMS2, MSH2 and MSH6, the probability of accumulating mutations in microsatellite regions increases exponentially [2,3]. Cancers with dMMR are thus often hypermutated, clustering mutations in microsatellite. This state is called microsatellite instability (MSI) [4]. Of note, dMMR and MSI show a high consistency (>95%), so they are almost interchangeably used [5]. For old terminologies, MSI was divided into high (MSI-H), low (MSI-L), and stable (MSS) [6]. Current recommendations and guidelines have clarified that MSI-L belongs to MSS, thus indicating that such terms should be abandoned [4]. Thus, the status of microsatellite is now classified into a dichotomous manner in stable (MSS) vs. instable (MSI).

MSI is the hallmark of hereditary nonpolyposis colorectal cancer syndrome (HNPCC), also called Lynch syndrome, and is found in various proportions of sporadic cancers occurring in endometrial, gastric, urothelial, central nervous system and especially colorectal [7,8,9]. United State Food and Drug Administration (FDA) approved immunotherapy in patients with advanced cancers harboring MSI/dMMR [10,11]. Clinical trials have shown improved advantages of immune checkpoint inhibitors in various solid tumors [12]. MSI/dMMR became the first pan-cancer and agnostic marker for the prediction of therapeutic response [13]. For these reasons, adequate validation of MSI is important for patient care, and demand for testing in the clinic has increased substantially. Currently, primary methods to test MSI status are immunohistochemistry (IHC) for DNA mismatch repair proteins, polymerase chain reaction (PCR)-based molecular testing, and next-generation sequencing (NGS), which is emerging as a novel but highly reliable option. However, current methods for the detection of MSI suffer from some limitations: they are labor-intensive, are subjectively interpreted, and have considerable costs [14,15]. Due to these shortcomings, many patients are still not tested, even though MSI universal testing is warranted in clinical practice [16]. To overcome these limitations, new technologies and systems are urgently needed.

It has been acknowledged that the genetic landscape could influence tumor histo-morphology [17]. For example, in the pancreas, tumors harboring MSI/dMMR usually show a typical histology, such as medullary and mucinous-colloid [18,19]. Of note, there were attempts to predict MSI status based on histomorphological features on hematoxylin-eosin (H&E) slides, which is accessible through daily routine work [20,21,22,23,24]. However, such attempts to predict MSI by histomorphology by humans have not achieved enough accuracy to replace existing methods. Recently, with the rapid development of artificial intelligence (AI), the ability to identify histomorphological patterns or characteristics for recognizing tumor molecular subtypes and for predicting the prognosis of diseases has been advanced [25,26,27,28,29,30]. Compared to humans, AI can detect subtle morphologic features that cannot be detected by the human eye. Since even these minor features can be detected by AI, it would show much more superior accuracy in predicting MSI status than humans [31]. In particular, a subtype of machine learning called deep learning is able to integrate multiple steps of computation to execute complex tasks [32]. With the development of deep learning technology over the past few years, its application into the medical and biomedical field is increasing exponentially [33]. Among deep learnings, convolutional neural networks (CNNs) are a specific type of deep neural network mostly applied in the field pathology image classification system. CNNs are inspired by the visual cortex structure of the human brain [32]. The original scanned image itself cannot be used for AI, and has to be processed for image analysis. CNNs consist of two parts, a neural network consisting of a combination of “convolution” and “pooling” layers in the front (feature extraction), and a neural network with a classification layer (multilayer perceptron) structure in the back. Conceptually, the convolution layer creates a newly modified version of images using many different types of filters (e.g., filters emphasizing contrast, filters highlighting edges of objects) The pooling layer summarizes the feature of images created by the convolution layer [34]. Repeating the preceding process, it creates many small images with different individual features from a single H&E slide image. Low-level features (e.g., lines, edge) in an early layer in the neural network are arranged so that deeper layers represent higher-level image features (e.g., motif, object) [35]. After repeating the convolution and pooling processes, fully connected layers (classification layer) are generated, and through this layer, the output is created. The output value is compared with the “true” label (reference standard) designated in the data, and if wrong, its filter is adjusted to improve the prediction accuracy [36]. Several studies on MSI/dMMR prediction through deep learning have shown promising results, but a comprehensive evaluation of their achievements is still lacking. Furthermore, most of the studies have been conducted with colorectal cancer, and only a few studies have been conducted on the rest of the tumors. To review the research flow of the MSI/dMMR prediction on the entire cancer types and to set a guide for future work, a wide and in-depth literature review of the studies so far is needed. In this study, we systematically review these studies, their reliability, and the potential risks of bias, while also discussing future perspectives.

## 2. Materials and Methods

This systematic review follows the Preferred Reporting Items for Systematic Re-view and Meta-Analyses (PRISMA) statement [37] (Table S1).

### 2.1. Inclusion and Exclusion Criteria

All studies published until 30 September 2021 using deep learning for predicting microsatellite instability on histopathologic slides are included. Exclusion criteria were: (1) Not using whole slide images of human tissue slides, (2) Not related to studies for microsatellite instability, (3) Not published in the English language, (4) Narrative re-view articles, case reports and letters to editors are also excluded.

### 2.2. Data Sources and Literature Search Strategy

Two investigators (J.H.P and J.I.S) independently searched PubMed, Embase to identify studies up to 30 September 2021. The search terms used in PubMed were as follows: (Artificial intelligence OR Machine learning OR Deep learning OR Computer-assisted OR Digital image analysis) AND (Microsatellite instability OR MSI OR MMR OR mismatch repair). A similar search was conducted in Embase. Manual selection of relevant articles through checking references of key articles was done additionally.

### 2.3. Study Selection and Data Extraction

**Figure 1:**
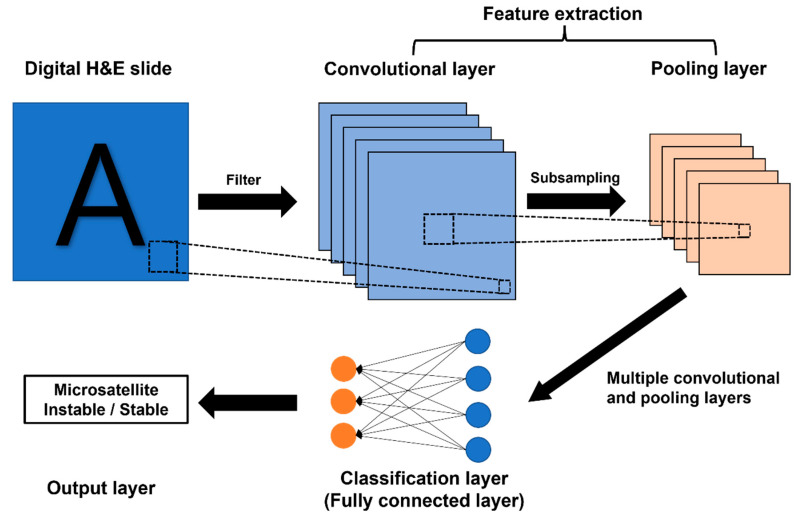
A simplified version of convolutional neural networks (CNNs) workflow in digital pathology. The scanned H&E slide image passes through filters that detect specific features (e.g., lines, edges). Pooling layers summarize features from convolution layers. After a series of convolution and pooling layers, fully connected layers (classification layer) are generated, and through this layer output is created.

After removal of duplicates, two investigators (J.H.P and B.J.L) screened the articles independently according to inclusion/exclusion criteria. (Figure 1) Any discord was discussed until an agreement was reached. For each article, information about authors, year of publication, type of model for AI, number of patients/images, cohort (training set, validation set), type of organ, performance outcome (area under the curve (AUC) and accuracy), methods for MSI/dMMR testing (ground truth/reference standard) were extracted. One author (J.H.P.) extracted data from each study and a second independent author (J.I.S.) validated the extracted data. Finally, all extracted data were reported and summarized in Table 1. The quality of each article was evaluated by Quality Assessment of Diagnostic Accuracy Studies (QUADAS-2) by two independent investigators (J.H.P. and B.J.L.) and summarized in Table S2 [38]. Only officially published articles are included in the QUADAS-2 evaluation.

## 3. Results

### 3.1. Search Results

**Figure 2:**
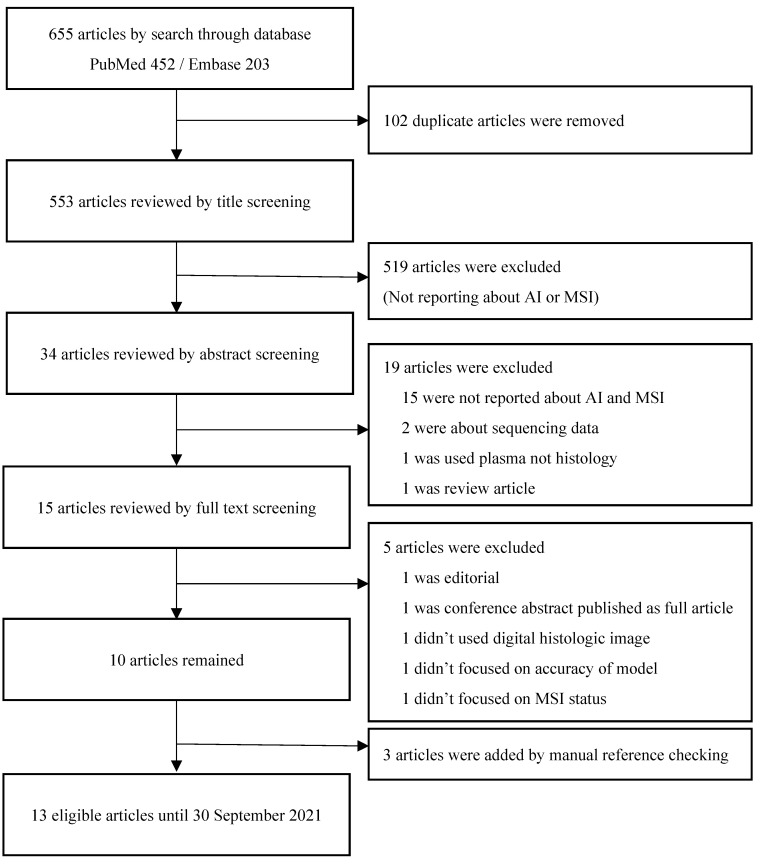
Flow chart of literature search.

The result of search yielded 553 non-duplicated articles. After excluding 538 articles based on title/abstract screening, 15 articles were retrieved for full text review. Five articles were excluded after the full text review and three were added manually by manual reference checking, and 13 articles were finally selected for systematic review (Figure 2) [39,40,41,42,43,44,45,46,47,48,49,50,51].

### 3.2. Predicting MSI/dMMR Status by AI-Based/Deep Learning—Approaches

**Figure 3:**
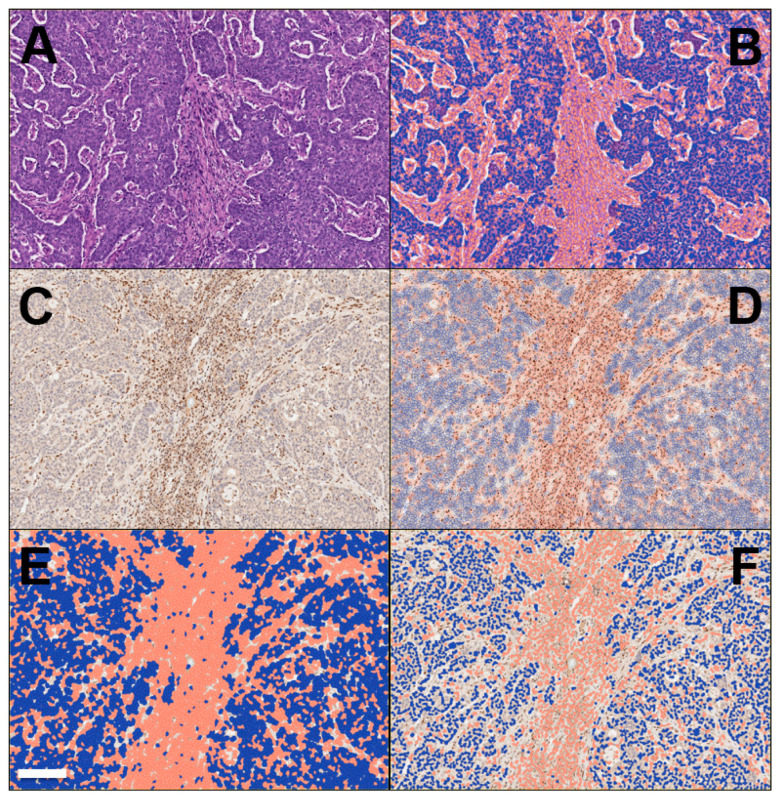
Representative workflow of the digitalization of a case of colon cancer with microsatellite instability is provided here (provided by Claudio Luchini, co-author). (**A**) Cancer area: tumor cells and peri-tumor cells, including stromal cells and immune cells, are here shown. This is the point of the start of the analysis on a slide stained with hematoxylin-eosin (original magnification: 10×). (**B**) The digitalized system is able to separate cancer cells (here colored in blue) from non-cancer cells (red). (**C**) The immunohistochemistry for mismatch-repair proteins can be also taken into account in this process. This figure represents MSL-staining, showing the loss of the protein into the neoplastic component, while its expression in retained in non-tumor cells (original magnification: 10×, same field of hematoxylin-eosin). (**D**) The digitalized system is able to interpret the results of immunohistochemistry, based on a deep learning approach. In this step, the system shows its ability to separate cancer cells (here colored in blue) from non-cancer cells (brown). (**E**,**F**) Since the immunohistochemistry for mismatch-repair proteins is a nuclear staining, for finalizing its interpretation the system here shows its ability in the detection and analysis of only cell nuclei, with tumor cells in blue and non-tumor cells in red (**E**,**F**; different resolution of analysis, which can be adapted based on staining patterns and the difficulty of their interpretation). Scale bar represents 200 μm.

No studies used prospectively collected data. In the vast majority of cases (11/13), studies were focused on colorectal cancer, alone or in combination with other cancer types (stomach, endometrium) (Table 1). Three studies investigated endometrial adenocarcinoma and four studies investigated gastric adenocarcinoma. Except for one study by Echle et al. [46], the rest of the studies used only The Cancer Genome Atlas (TCGA) data or included TCGA as part of the study cohort. Methods for dividing the cohorts into training and test sets were different across the studies. Three studies used K-fold cross-validation and seven studies randomly split data into a training set and test set. One conference abstract didn’t specify the method [42]. Two studies attempted to compare performance using both methods. Regarding the methodology for assessing MSI/dMMR status (as reference standard), nine studies used MSI-based PCR, three used IHC, and three used NGS to establish a ground truth. Three unpublished studies (conference paper, abstract) did not disclose the specific method for MSI/dMMR assessment. Since each study collected various data groups and created a research cohort, the method of setting the reference standard was different within one study. For example, in the study of Kater et al. [41], PCR was used in the TCGA cohort and Darmkrebs Chancen der Verhütung durch Screening (CRC prevention through screening study abbreviation in German; DACHS) cohort, but Kangawa cancer center hospital (KCCH) data used IHC. And also, in the case of the adoption of the same methodology, some discrepancies were present among different studies: this was recoded for MSI-PCR, since some studies used non-standardized methods. Indeed, the DACHS data group use 3-plex PCR for its confirmation and the United Kingdom-based Quick and Simple and Reliable trial (QUASAR) and the Netherlands Cohort Study (NLCS), data group use 2-plex PCR, where the gold standard calls for at least 5 markers [4]. This was the same for IHC as for NLCS, which used an IHC panel with only two antibodies and without a PCR for confirmation. Nine out of 13 studies measured MSI/dMMR prediction-performance using an external validation cohort. As for the form of reporting the performance value, one conference paper was published as accuracy, and the rest of the study was reported as AUC. One study which reported the performance value of the model as accuracy shows up to 98.3% of accuracy for colorectal cancer and up to 94.6% for endometrial carcinoma. As for AUC, studies using colorectal cancer tissue showed a higher performance (highest standard of 0.972) compared to stomach and endometrial tumors (highest standard of 0.81 and 0.82 respectively) which is a relatively low but satisfactory performance. Individual studies used various deep learning models for prediction (Table 1, Figure 3). Of the 12 studies (one study did not reveal the exact model), five studies used the ResNet-based model, accounting for the largest number, followed by the ShuffleNet model used in three studies. Inception-V3, MSInet, and InceptionResNetV1 were used in two, one, and one studies, respectively.

The model with the highest performance for predicting MSI in colorectal cancer was developed by Lee et al. [50]. In their study, Inception-V3 was trained on a cohort composed of images from TCGA and Saint Mary’s Hospital (SMH). When this trained model was tested on an internal validation cohort (TCGA), AUC was 0.892 and AUC tested on another internal cohort (SMH dataset) was 0.972, which is the highest value reported in the included studies. The authors also developed another model only trained on the TCGA dataset. When this model was tested to external validation cohort (SMH dataset), the performance of this model dropped by 0.787. When tested on the internal cohort (TCGA), AUC was 0.861.

As for endometrial carcinoma, the strongest model for MSI prediction was developed by Hong et al. [51]. In this study, the authors trained the model using the TCGA and Clinical Proteomic Tumor Analysis Consortium (CPTAC) dataset. When this model was tested on an internal cohort, it achieved an AUC of 0.827. However, when tested on an external cohort, performance was dropped by 0.667. The strongest performance outcome for stomach cancer was demonstrated by Valieris et al. [47]. They trained Resnet-34 using a TCGA dataset and when tested on an internal cohort, the model achieved an AUC of 0.81. A similar result was shown by Kater et al. [41].

Among the individual studies, Echle et al. [46] used the largest amount of data for model development. The MSIDETECT consortium, which consists of TCGA, DACHS, QUASAR, and NLCS was used as a training material. They demonstrated a good performance value for predicting MSI on both internal and external validation cohorts (0.92 for the internal cohort and 0.96 for the external cohort).

### 3.3. Assessment of the Risk of Bias and Applicability

When the overall risk of bias and applicability was measured with the QUADAS-2 tool, most studies had one or more high risk factors (Table S2). For the “patient selection” domain, a high risk of bias was detected in three studies (30%). Studies using only TCGA data without adding other data groups to increase data diversity were judged at high risk. For the “index test” domain, six studies were found to be at high risk of bias. This was due to the absence of an external validation cohort and for not using K-fold cross validation in the data split process. For the “reference standard” domain, high risk was applied to those studies that used IHC only for MSI/dMMR assessment. Based on this criterion, two other studies have been judged to be in the high risk group (20%). At last, no high risk of bias study was identified in “flow and timing” domain.

## 4. Discussion

Recent studies have shown that AI-based deep learning models can predict molecular alterations through histopathological features of digital slides [25,44,52]. One of the most important biomarkers under investigation is MSI/dMMR, which is the most widely validated biomarker [46]. Current methodologies of MSI/dMMR assessment suffer from some limitations including potential low-reproducibility of IHC and high costs of direct molecular tests (MSI-based PCR and NGS), the latter being available only in tertiary medical institutions [53]. Some attempts to determine MSI/dMMR using clinical data and histopathological features in patients, especially those with colorectal cancer, have been made prior to the development of AI-based deep learning technologies. Some studies focused on colorectal cancer reported that MSI/dMMR tumors have peculiar histomorphological features (e.g., mucinous, signet-ring or medullary morphology, poor differentiation, tumor infiltrating lymphocytes, peritumoral lymphoid reaction, Crohn’s-like lymphoid reaction, signet ring cells) and clinical findings (lower age, right-colonic tumor location) compared to MSS-neoplasms [20,22,23,54]. A similar study with overlapping results was also conducted in endometrial adenocarcinoma [24]. MSI status was predicted by integrating information on histological features and clinical data [22,23,55,56]. However, skilled pathologists also had to spend a lot of time to confirm each histological findings and interobserver variability may affect the reliability of results [57,58,59]. Eventually, this method cannot replace the existing detection system. However, these attempts have established the premise of predicting MSI from the morphological features and now AI-based deep learning technologies could be of great help in overcoming existing issues. In addition to some of the morphological findings related to MSI revealed in previous human-based studies, subtle morphological features that humans can’t find are identified and collectively judged by AI. However, none of the individual studies mentioned histological characteristics that were thought to be related to MSI. This is because, due to the nature of deep learning models, researchers can develop models through training data and obtain prediction results through tests data, but they can’t know what kind of thinking flow the model itself makes decisions through. The performance of the deep learning algorithm is determined by various variables such as type of network architecture, sample preparation, size of cohorts, and method of defining the ground truth method (reference). Among them, the use of a diverse large amount of data has a great impact on performance: the higher the quality of images used for training, the better will be tumor detection and molecular subtyping [46,59]. However, unlike non-medical fields, these images are tied to legal and ethical regulation as patient personal information, making it difficult to build data available for model development [60]. Due to these limitations, there are few available datasets such as TCGA, which is adopted as a validation cohort in several studies. In a recent investigation, the generative model was used to create artificial histopathology images that was difficult to distinguish compared to real images [61,62,63]. In a study by Krause et al. [49], a cohort containing synthetic images used for a training model showed non-inferior results in performance compared to models trained with real images only. This study indicated new alternatives also to overcome data acquisition limitations.

Multi-national and multi-institutional datasets are essential for developing generalizable models which reflect differences between diverse regions and ethnicities around the world [64,65]. In a recent study by Kater et al. [41], the model trained with TCGA data showed a poor performance in a Japanese cohort (KCCH), and similar results were identified in remaining studies. Cao et al. [45] also showed a low level of AUC when a model trained on TCGA data was applied to the Asian group, but the inclusion of the Asian group in the training set showed an improved performance. Thus, for deep learning technology, it is necessary to quantitatively ensure that there is a lot of data (so-called big data) reflecting the actual real world patient group, so that algorithms can learn through reliable training materials. All of these findings and considerations call for taking into account demographic and representative data of different ethnic groups for building robust AI-based systems to be used in clinical practice.

It is also crucial to secure an independent external validation set in model evaluation [66], distinguishing training groups and validation-groups. This should be seen as an important tool for guaranteeing a high level of reliability for AI-based systems, the assumption “garbage-in, garbage-out” always being valid.

Reporting the reference standard of individual studies also did show some variations. According to current guidelines [4], the suggested methods for MSI/dMMR detection are as follows: (1) IHC alone, but only for those tumors strictly belonging to the spectrum of HNPCC (Lynch) syndrome; (2) MSI-based PCR based on at least five of the Bethesda markers and including mandatorily BAT25 and BAT26; (3) NGS, above all for tumors out of HNPCC spectrum or in the case of limited neoplastic material (e.g., biopsy, cytological samples). Not only there is a variation, but if MSI/dMMR status was determined with 2-plex PCR or IHC out of HNPCC spectrum, not-negligible doubts may arise about the reliability of the reference standard. Given that labelling data set in a correct manner is fundamental for evaluating the performance of a given model, the existence of such variability for defining the reference standard poses limitations and concerns in evaluating the overall performance of the model.

QUADAS-2 is an assessment tool for systematically evaluating the risk of bias and applicability of individual studies of diagnostic accuracy. It has been used as a quality assessment tool in this review, as per recent guidelines. Over the past decade, research on AI-based diagnostic tests has been centered on AI research in the healthcare field. Of note, more than 90% of health-related AI systems that have received regulatory evaluation from the US Food and Drug Administration are related to the diagnostic field [27]. Although AI-based diagnostic research is carried out extensively, the existing QUADAS-2 tool for evaluating diagnostic research may be not sufficient to reflect the specificity of such a heterogeneous field, so it may be of help to consider the modified quality’s evaluation tools. Notably, an AI-specific tool (QUADAS-AI) is currently under development and is expected to be published soon. The publishment of a specific tool will help in a closer evaluation of each diagnostic research in the AI field. 

Although the diagnostic performance of individual studies is reported to be excellent, it is unclear whether it is immediately applicable to clinical practice due to the different risk factors that have been identified through QUADAS-2 and also due to variations between each individual study mentioned above.

## 5. Conclusions

Given that immunotherapy is at the center of the paradigm of cancer treatment in these last years, AI-based/deep learning technologies able to predict MSI status are expected to have a great impact into the diagnostic workflow of oncology and related areas, playing important roles as a standardized diagnostic tool. The presence of not-negligible limitations, however, calls for a direct molecular confirmation (MSI-based PCR or NGS) in those cases that are positive at evaluation by AI-based systems.

## Figures and Tables

**Table 1:** Comparison of studies regarding detection of MSI/dMMR from histology slides using deep learning.

Author/Year	Organ (% by Training Cohort)	Neural Network	Training Cohort	Type of Internal Validation	External Validation	Test Cohort(s) with AUC (95% CI) or Accuracy	Methodology for MSI Analysis
Zhang et al., * 2018 [39]	Colorectum (51.3%) UCEC (48.7%) Stomach ^†^	Inception-V3 without adversarial training	TCGA CRC (*n* = 585 WSIs)	Random split	Yes	TCGA CRC Accuracy: 98.3% TCGA UCEC Accuracy: 53.7%	-
			TCGA CRC (*n* = 585 WSIs) TCGA UCEC (*n* = 556 WSIs)			TCGA CRC Accuracy: 72.3% TCGA UCEC Accuracy: 84.2% TCGA STAD (*n* = 209 WSIs) Accuracy: 34.9%	
		Inception-V3 with adversarial training				TCGA CRC Accuracy: 85.0% TCGA UCEC Accuracy: 94.6% TCGA STAD (*n* = 209 WSIs) Accuracy: 57.4%	
Klaiman et al., * 2019 [40]	Colorectum	N/A	Roche internal CRC80 dataset (*n* = 94 pts)	Random split	No	Roche internal CRC80 dataset: 0.9	-
Kater et al., 2019 [41]	Stomach (19.2%)	ResNet-18	TCGA STAD FFPE (*n* = 216 pts)	Random split	Yes	TCGA STAD FFPE (*n* = 99 pts): 0.81 (0.69–0.90) DACHS FFPE (*n* = 378 pts): 0.60 (0.48–0.69) KCCH FFPE (*n* = 185 pts): 0.69 (0.52–0.82)	TCGA: PCR DACHS: PCR ^1^ KCCH: IHC
	Colorectum (23.1%)		TCGA CRC FFPE (*n* = 260 pts)			TCGA CRC FFPE (*n* = 100 pts): 0.77 (0.62–0.87) DACHS FFPE (*n* = 378 pts): 0.84 (0.720–0.92)	
	Colorectum (23.8%)		TCGA CRC Frozen (*n* = 269 pts)			TCGA CRC Frozen (*n* = 109 pts): 0.84 (0.73–0.91) DACHS FFPE (*n* = 378pts): 0.61 (0.50–0.73)	
	UCEC (33.9%)		TCGA UCEC FFPE (*n* = 382 pts)		No	TCGA UCEC FFPE (*n* = 110 pts): 0.75 (0.63–0.83)	
Pressman et al., * 2020 [42]	Colorectum	ResNet18	TCGA (*n* = 360 WSIs)	-	Yes	TCGA: 0.79 Gangnam sev (*n* = 170 WSIs): 0.76	-
Schmauch et al., 2020 [43]	Colorectum (62.8%)	HE2RNA with ResNet50	TCGA CRC FFPE (*n* = 465 pts)	Three-fold cross validation	No	TCGA CRC FFPE: 0.82	PCR
	Stomach (37.2%)		TCGA STAD FFPE (*n* = 276 pts)			TCGA STAD FFPE: 0.76	
Kather et al., 2020 [44]	Colorectum	ShuffleNet	TCGA CRC FFPE (*n* = 426 pts)	Three-fold cross-validation	Yes	DACHS FFPE (*n* = 379 pts): 0.89 (0.88–0.92)	TCGA: PCR DACHS: PCR ^1^
Cao et al., 2020 [45]	Colon	ResNet-18	TCGA-COAD Frozen (Total number including test cohort: 429 WSIs)	Random split	Yes	TCGA-COAD: 0.8848 (0.8185–0.9512) Asian-CRC FFPE (*n* = 785 WSIs): 0.6497 (0.6061–0.6933)	TCGA-COAD: NGS ^2^ Asian-CRC; PCR
			TCGA-COAD Frozen (90%) + Asian-CRC FFPE (10%)	-	No	Asian-CRC FFPE (*n* = 785 WSIs): 0.8504 (0.7591–0.9323)	
			TCGA-COAD Frozen (30%) + Asian-CRC FFPE (70%)	-	No	Asian-CRC FFPE (*n* = 785 WSIs): 0.9264 (0.8806–0.9722)	
Echle et al., 2020 [46]	Colorectum	ShuffleNet	MSIDETECT CRC (*n* = 6406 pts)	Random split	Yes	MSIDETECT CRC: 0.92 (0.90–0.93)	DACHS: PCR TCGA: PCR QUASAR and NLCS: IHC ^3^ YCR-BCIP: IHC
				Three-fold cross validation		MSIDETECT CRC: 0.92 (0.91–0.93) YCR-BCIP-RESECT (*n* = 771 pts): 0.96 (0.93–0.98) YCR-BCIP-BIOPSY (*n* = 1531 pts): 0.78 (0.75–0.81)	
			YCR-BCIP-BIOPSY (*n* = 1531 pts)	Three-fold cross validation	No	YCR-BCIP-BIOPSY: 0.89 (0.88–0.91)	
Valieris et al., 2020 [47]	Stomach	Resnet-34	TCGA-STAD FFPE (Total number including test cohort: 369 pts)	Random split	No	TCGA-STAD FFPE: 0.81 (0.689–0.928)	NGS ^4^
Yamashita et al., 2021 [48]	Colorectum	MSInet	Stanford dataset (*n* = 85 pts)	Random split	No	Stanford dataset (*n* = 15 pts): 0.931 (0.771–1.000)	Stanford dataset: IHC/PCR TCGA: PCR
				Four-fold cross-validation	Yes	Stanford dataset (*n* = 15 pts): 0.937 TCGA (*n* = 479 pts): 0.779 (0.720–0.838)	
Krause et al., 2021 [49]	Colorectum	ShuffleNet	TCGA FFPE (*n* = 256 pts)	Random split	No	TCGA FFPE (*n* = 142 pts): 0.742 (0.681–0.854)	PCR
Lee et al., 2021 [50]	Colorectum	Inception-V3	TCGA FFPE (*n* = 470,825 patches) SMH FFPE (*n* = 274 WSIs)	10-fold cross validation	No	TCGA FFPE: 0.892 (0.855–0.929) SMH FFPE: 0.972 (0.956–0.987)	TCGA: PCR SMH: PCR/IHC
			TCGA FFPE (*n* = 470,825 patches)		Yes	TCGA FFPE: 0.861 (0.819–0.903) SMH FFPE: 0.787 (0.743–0.830)	
			TCGA Frozen (*n* = 562,837 patches)		No	TCGA Frozen: 0.942 (0.925–0.959)	
Hong et al., 2021 [51]	UCEC	InceptionResNetV1	TCGA and CPTAC (Total number including test cohort: 456 pts)	Random split	Yes	TCGA and CPTAC: 0.827 (0.705–0.948) NYU set: 0.667	TCGA: PCR CPTAC: NGS ^5^

AUC, Area Under the Curve; UCEC, Uterine Corpus Endometrial Carcinoma; TCGA, The Cancer Genome Atlas study; CRC, ColoRectal Cancer; WSI, Whole Slide Images; STAD, STomach ADenocarcinoma; pts, patients; FFPE, Formalin-Fixed Paraffin-Embedded; DACHS, Darmkrebs: Chancen der Verhütung durch Screening (CRC prevention through screening study abbreviation in German); KCCH, Kangawa Cancer Center Hospital (Japan); Stanford dataset, Stanford University Medical Center (USA) Gangnam sev, Gangnam Severance Hospital (South Korea); COAD, COlonic ADenocarcinoma; MSIDETECT: A consortium composed of TCGA, DACHS, the United Kingdom-based Quick and Simple and Reliable trial (QUASAR), and the Netherlands Cohort Study (NLCS); YCR-BCIP: Yorkshire Cancer Research Bowel Center Improvement Programme; SMH, Saint Mary’s Hospital (South Korea); CPTAC, Clinical Proteomic Tumor Analysis Consortium; NYU, New York University hospital. * Conference paper or abstract not officially published. ^†^ The stomach cancer cohort was used only in test cohort. ^1^ 3-plex PCR (BAT25, BAT26, CAT25) ^2^ MSI sensor algorithm ^3^ 2-plex IHC ^4^ Mutation signature ^5^ Mutation load, MMR gene mutation status, MSI sensor score, MSMuTect score, and MLH1methylation.

## Data Availability

Not applicable.

## Supplementary Materials

The following supporting information can be downloaded at: https://www.mdpi.com/article/10.3390/ijms23052462/s1.

## Abbreviations

MSI: Microsatellite instability; dMMR: Defective mismatch repair; STR: Short tandem repeats; SSR: Simple sequence repeats; IHC: Immunohistochemistry; PCR: Polymerase chain reaction; NGS: Next-generation sequencing; AI: Artificial intelligence; AUC: Area under the ROC curve; UCEC: Uterine corpus endometrial carcinoma; TCGA: The cancer genome atlas; CRC: Colorectal cancer; WSI: Whole slide images; STAD: Stomach adenocarcinoma; FFPE: Formalin-fixed paraffin-embedded; DACHS: Darmkrebs Chancen der Verhütung durch Screening (CRC prevention through screening study abbreviation in German); KCCH: Kangawa cancer center hospital; COAD: Colonic adenocarcinoma; YCR-BCIP: Yorkshire cancer research bowel center improvement programme; SMH, Saint Mary’s hospital; CPTAC: Clinical proteomic tumor analysis consortium; NYU: New York university hospital.
